# Multi-Robot Cooperative Simultaneous Localization and Mapping Algorithm Based on Sub-Graph Partitioning

**DOI:** 10.3390/s25092953

**Published:** 2025-05-07

**Authors:** Wan Xu, Yanliang Chen, Shijie Liu, Ao Nie, Rupeng Chen

**Affiliations:** School of Mechanical Engineering, Hubei University of Technology, Wuhan 430068, China; chenyanliang1025@163.com (Y.C.); 102310080@hbut.edu.cn (S.L.); nieao@hbut.edu.cn (A.N.); 2010551111@hbut.edu.cn (R.C.)

**Keywords:** multi-robot systems, multi-sensor fusion, distributed optimization, loop closure detection, collaborative SLAM

## Abstract

To address the challenges in multi-robot collaborative SLAM, including excessive redundant computations and low processing efficiency in candidate loop closure selection during front-end loop detection, as well as high computational complexity and long iteration times due to global pose optimization in the back-end, this paper introduces several key improvements. First, a global matching and candidate loop selection strategy is incorporated into the front-end loop detection module, leveraging both LiDAR point clouds and visual features to achieve cross-robot loop detection, effectively mitigating computational redundancy and reducing false matches in collaborative multi-robot systems. Second, an improved distributed robust pose graph optimization algorithm is proposed in the back-end module. By introducing a robust cost function to filter out erroneous loop closures and employing a subgraph optimization strategy during iterative optimization, the proposed approach enhances convergence speed and solution quality, thereby reducing uncertainty in multi-robot pose association. Experimental results demonstrate that the proposed method significantly improves computational efficiency and localization accuracy. Specifically, in front-end loop detection, the proposed algorithm achieves an F1-score improvement of approximately 8.5–51.5% compared to other methods. In back-end optimization, it outperforms traditional algorithms in terms of both convergence speed and optimization accuracy. In terms of localization accuracy, the proposed method achieves an improvement of approximately 32.8% over other open source algorithms.

## 1. Introduction

Compared to the rapid development of single-robot SLAM, the field of multi-robot SLAM still faces many unresolved challenges. Currently, research on multi-robot collaborative SLAM primarily focuses on extending single-robot SLAM algorithms, with researchers emphasizing aspects such as inter-robot communication, cooperative localization progress, operational efficiency, and map consistency [1,2]. Multi-robot systems, owing to their efficient collaboration, strong generalization capabilities, and flexible reconfigurability [3], have been widely applied in areas such as field search and rescue, area coverage, and environmental exploration [4]. During the execution of these tasks, collaborative simultaneous localization and mapping (CSLAM) is crucial. With the aid of CSLAM techniques, robots are able to cooperatively estimate their own poses and construct a global map in large-scale unknown environments, which is of great significance for enhancing the autonomy and intelligence of multi-robot systems [5].

## 2. Related Work

Loop closure detection is the process by which a robot recognizes that its current environment is identical to a previously encountered one and confirms that it has returned to a known location. It is a crucial component of multi-robot collaborative SLAM [6,7]. He et al. [8] introduced the Mesh-to-3D-Point cloud (M2DP) descriptor, which creates several 2D signatures by analyzing how points are spread out on a flat surface. The left and right singular vectors of the signature matrix use these signatures to construct a descriptor that represents 3D point clouds. However, in environments with multiple robots, differences in when and how each robot collects data can cause problems with M2DP descriptors, which negatively impacts the accuracy of matching point clouds and detecting when a robot returns to a previous location. Wohlkinger et al. [9] enhanced descriptor discriminability by combining angle, distance, and area shape functions. However, since the Ensemble of shape functions for a 3D object classification (ESF) descriptor requires high-dimensional statistical analysis of the shape features of point clouds, it has the high computational complexity, making it unsuitable for multi-robot SLAM systems with limited computational resources. Wang et al. [10] introduce the intensity scan context (ISC) descriptor, which generates a 2D matrix representation using the maximum intensity value within a subspace and integrates geometric and intensity features to characterize the environment. However, as the descriptor relies on LiDAR intensity information, significant variations in intensity values among different sensors pose challenges for standardized heterogeneous multi-robot SLAM systems. Article et al. [11] prioritize loop closures for computations based on the underlying pose graph, proximity to known landmarks, and point cloud features. Chen et al. [12] suggested a strong method for choosing loop closures in map merging that works both within a robot and between robots, taking into account the consistency of pairs and the accuracy of movement to reduce errors from incorrect loop closures and noise in movement data.

In a multi-robot collaborative SLAM system, each robot estimates its trajectory based on its own measurements. When encountering other robots, it exchanges information to obtain relative poses, thereby optimizing its own pose. At this stage, all robot trajectories are integrated into a set of poses to be estimated, with the objective of maximizing the likelihood of measurement data. This transforms the maximum likelihood estimation problem into minimizing the sum of squared errors. However, this optimization problem involves non-convex constraints, making it computationally complex and highly sensitive to the accuracy of initial poses. Therefore, an efficient distributed SLAM optimization method is urgently needed to reduce the difficulty of solving non-convex constraints and decrease reliance on precise initial poses [13,14]. Patel et al. [15] proposed visual–inertial SLAM for Centralized Collaboration-G (COVINS-G), a general backend algorithm built upon the COVINS framework, which enables compatibility between a server-side backend and arbitrary visual–inertial odometry front-ends. It utilizes multi-camera relative pose estimation algorithms to process 2D image data for computing loop closure constraints. However, in highly occluded environments with sparse feature points, its loop closure detection accuracy is affected, leading to a decline in mapping precision. Cunningham et al. [16] introduced the fully distributed SLAM using the constrained factor graphs (DDF-SAM) method, which employs an extended smoothing and mapping approach for decentralized data fusion. It incorporates modules for local optimization, communication, and neighborhood graph optimization, offering scalability in computational cost and communication bandwidth while maintaining robustness against node failures and network topology changes. However, in communication environments with high latency and severe packet loss, data transmission and synchronization between modules become problematic, resulting in untimely and inaccurate map information fusion. Han et al. [17] proposed a distributed multi-robot SLAM framework that uses sliding-window-based optimization to reduce computational load and handle inter-robot loop constraints. It facilitates map consistency and bandwidth efficiency by transmitting 2.5D grid submaps based on keyframes. However, in dynamic environments with rapidly moving objects and frequent occlusions, the keyframe-based map construction and update mechanism may fail to adapt in time, causing deviations between the generated map and the actual environment. Chang et al. [18] developed a multi-robot SLAM algorithm that constructs a graph-like topological map, where map fusion is achieved by adding edges, and relative robot poses are optimized through edge estimation. This approach exhibits scalability concerning the number of robots and the environmental size. However, in environments with complex topology and multiple similar regions, topological map construction is prone to erroneous connections and misclassifications, ultimately compromising map accuracy. Tian et al. [19] proposed a distributed pose graph optimization algorithm based on sparse semidefinite relaxation, which can recover the global optimal solution under moderate noise while being communication-efficient and privacy-preserving. However, under poor network conditions, such as sparse network topology or increased latency, the algorithm’s performance deteriorates. The two-stage Gauss–Seidel method proposed by Choudhary [20] for pose recovery suffers from significant sensitivity to initialization and lacks guarantees for convergence to first-order critical points. To address this, Fan [21] and colleagues employed a majorization–minimization (MM) approach to solve the distributed pose graph optimization (PGO) problem, successfully ensuring convergence. Some studies have incorporated robot dynamic models and designed distributed consensus algorithms using feedback control theory. A representative example is the Kimera-Multi [22] algorithm, which can identify and reject incorrect loop closures under limited communication bandwidth, enabling the construction of a globally consistent 3D semantic mesh model. However, existing distributed PGO methods are mostly based on the sequential partitioning of local robot pose information, without fully leveraging the structural characteristics of the pose graph, which limits the optimization performance.

The centralized processing of all robot pose information in the backend optimization leads to exponentially increasing computational complexity and imposes stringent requirements on the accuracy of initial poses. Initial errors tend to accumulate, adversely affecting overall algorithm performance. Graph partitioning is critical in distributed PGO. The commonly used spectral decomposition method [23] often suffers from high computational complexity or neglects balance constraints, making it unsuitable for large-scale graph segmentation. In contrast, heuristic [24] and evolutionary algorithms [25] are more mature, and multilevel graph partitioning algorithms offer significant advantages in terms of computational speed and partition quality. Xu [26] and colleagues proposed an online streaming graph partitioning method and the Balanced Distributed Pose Graph Optimization (BDPGO) framework for swarm robotics, which demonstrate certain robustness and can handle robot failures. However, they are sensitive to dynamic changes in the graph and rely heavily on the input order of data, failing to effectively address the communication and computational inefficiencies caused by poor partitioning. Existing methods have their respective strengths and limitations, highlighting the need for more effective graph partitioning strategies to balance the subproblem dimensionality, reduce communication overhead, and enhance the overall performance of distributed pose graph optimization algorithms. Table 1 summarizes the advantages and disadvantages of traditional algorithms.

In summary, existing multi-robot collaborative SLAM techniques still have certain limitations in both front-end loop closure detection and back-end optimization. Current algorithms often incorporate loop closure frames with low correlation into the computation, limiting their recognition capability in front-end loop closure detection. This increases the communication data volume, exacerbates the computational load on robots, and strains both communication and computational resources. In the back-end optimization, when applied to large-scale multi-robot systems and complex noisy environments, existing methods suffer from suboptimal graph partitioning, slow algorithm convergence, and low optimization accuracy. Overall, current approaches still require improvements in real-time performance and environmental adaptability. This paper addresses the shortcomings of existing technologies by proposing a multi-robot collaborative SLAM algorithm based on subgraph partitioning. First, a global matching and loop closure candidate selection strategy is introduced in the front-end loop closure detection module, combining laser point clouds and visual features to achieve cross-robot loop closure detection, effectively alleviating issues such as high computational redundancy and mismatching rates in multi-robot collaboration. Second, in the back-end optimization module, an improved distributed pose graph optimization algorithm is proposed. By introducing a cost function to eliminate anomalous loop closure data and adopting a subgraph optimization approach during the iterative optimization process, the algorithm improves convergence speed and solution quality, thereby reducing the uncertainty of multi-robot pose associations. Finally, through simulations and experiments, the efficiency and accuracy of the proposed multi-robot collaborative SLAM algorithm based on subgraph partitioning are validated.

This paper presents the following contributions and innovations:

1. To address the issue of loop closure association among multiple robots, this paper proposes a communication-constrained inter-robot loop closure detection algorithm based on algebraic connectivity maximization. A sparse budgeted selection algorithm driven by algebraic connectivity maximization is employed to identify candidate loop closures, effectively avoiding the processing of excessive redundant measurements and reducing both communication and computational burdens.
2. To address the multi-robot collaborative localization optimization problem, this paper proposes an improved distributed pose graph optimization algorithm. The algorithm utilizes the measurements from the loop closure detection module and the pose estimates from the odometry to compute consistency metrics, thereby enabling the removal of outliers. By performing iterative optimization on the pose graph in subgraph blocks, the algorithm improves the convergence speed and solution quality, ultimately providing accurate pose estimates for each robot in the global coordinate system.

## 3. Methodology

**Figure 1:**
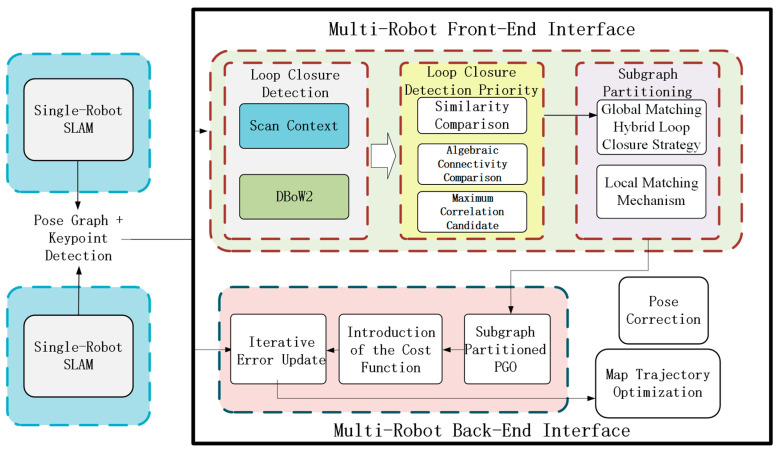
System framework of multi-robot collaborative SLAM.

Figure 1 illustrates the system framework of the proposed multi-robot collaborative SLAM. First, the single-robot SLAM module outputs pose estimates and keypoint detection information, which serve as the fundamental data for the multi-robot SLAM system. These data are then processed by the multi-robot front-end interface, where a loop closure detection algorithm calculates similarity scores. A Laplacian matrix is introduced to compute the maximum associated candidate edge set for each robot, and the most connected candidate edges are selected to form subgraphs. The processed subgraphs are then fed into the multi-robot back-end interface. First, subgraph-based pose graph optimization (PGO) is performed by integrating single-robot pose estimates with inter-robot candidate edges. Next, a cost function is introduced to evaluate the measurement errors of each edge, followed by the iterative optimization of the pose estimates within the pose graph. Finally, the system outputs corrected poses, completing the optimization of each robot’s trajectory and map.

### 3.1. Robot Hybrid Loop Closure Detection Strategy

In the front-end of multi-robot collaborative SLAM systems, robots rely on transmitting keyframes to obtain relative poses. However, when computing loop closures, incorrect or loosely correlated loop frames are often introduced. This not only leads to the accumulation of errors in the robot’s pose estimation, causing the constructed map to deviate from the actual environment and impacting the system’s reliability and usability, but also consumes limited communication and computational resources. Therefore, this section proposes a loop closure detection algorithm under communication constraints based on maximizing algebraic connectivity. The algorithm utilizes the Laplacian matrix to select loop closures and applies a reasonable strategy to filter out correct loop closure edges while avoiding the processing of large amounts of redundant measurements. Additionally, subgraph partitioning is used to select the most connected loop closures for the robots, laying the foundation for multi-robot back-end pose graph optimization. The proposed algorithm integrates laser point clouds and visual features to perform cross-robot loop closure detection. The laser point cloud detection utilizes lidar to capture point cloud data, analyzing its features to identify environmental characteristics that assist in loop closure detection. The visual feature detection uses the camera to extract image feature points, representing them with descriptors and comparing the descriptors of image feature points at different times to detect loop closures. This multi-sensor fusion approach leverages the strengths of both lidar and cameras to enhance the accuracy of loop closure detection.

#### 3.1.1. Global Matching Hybrid Loop Closure Strategy

In traditional global matching, greedy algorithms [22] simply select the top A candidates with the highest similarity scores, where A is predefined by the user based on the robot’s communication and computational capacity. However, this approach may lead to candidate selections that are overly concentrated in high-similarity regions, resulting in redundancy. To address this issue, this section incorporates the concept of pose graph sparsification into the candidate prioritization process. By ranking candidate loop closure edges based on priority, the algorithm selects a subset of candidates that maximizes algebraic connectivity. This method ensures that the selected candidates are more evenly distributed across the pose graph, effectively reducing redundancy.

The multi-robot pose graph is defined to model the spatial relationships and measurement data among robots, and it is expressed as:

(1) $$X=\left(V,S\right)$$

where $V=\left(V_{1},\cdots ,V_{n}\right)$ represents the set of vertices, with n denoting the number of robots. $V_{i}$ corresponds to the set of pose vertices for the ith robot, where each vertex represents the robot’s pose at a specific time. These vertices encapsulate the observation information of robots at different time instances and serve as fundamental elements in constructing the pose graph. The set of edges $S=\left(S^{local},S^{\mathrm{global}}\right)$ consists of two types of edges. The local edge set $S^{\mathrm{local}}=\left(S_{1}^{\mathrm{local}},\cdots ,S_{n}^{\mathrm{local}}\right)$ represents intra-robot pose constraints, including odometry measurements and robot-specific loop closures. These edges capture the relative pose changes during a robot’s motion and its internal loop closures, which are crucial for maintaining the accuracy of local robot poses. The global edge set $S^{\mathrm{global}}=\left(S_{1}^{\mathrm{global}},\cdots S_{n}^{\mathrm{global}}\right)$ consists of inter-robot loop closure candidate edges, representing potential loop closures between different robot poses. These edges are the primary focus of subsequent optimization.

To evaluate the algebraic connectivity of the pose graph, the rotated weighted Laplacian matrix is introduced as L=D−A, where D is the degree matrix and A is the adjacency matrix. In multi-robot pose graph optimization, the adjacency matrix A represents the direct connectivity relationships between robot poses, while the degree matrix D reflects the connectivity degree of each robot pose. The Laplacian matrix L integrates both pieces of information and is used to analyze the overall structure and stability of the pose graph.

The degree matrix D is an n×n diagonal matrix, where each diagonal element $D_{ii}$ represents the degree of vertex i, defined as the number of edges connected to vertex i. It is expressed as:

(2) $$D=\left(\begin{array}{cccc} D_{11} & 0 & \cdots & 0\\ 0 & D_{22} & \cdots & 0\\ \vdots & \vdots & \ddots & \vdots \\ 0 & 0 & \cdots & D_{nn} \end{array}\right)$$

In the multi-robot pose graph, $D_{ii}$ represents the number of relative pose constraints between robot i and the poses of other robots. A larger $D_{ii}$ indicates that the robot has interactions with a greater number of other robots within the multi-robot system.

The adjacency matrix A is also an n×n matrix used to describe the connectivity relationships between vertices in the graph:

(3) $$A=\left(\begin{array}{cccc} A_{11} & A_{12} & \cdots & A_{1n}\\ A_{21} & A_{22} & \cdots & A_{2n}\\ \vdots & \vdots & \ddots & \vdots \\ A_{n1} & A_{n2} & \cdots & A_{nn} \end{array}\right)$$

Specifically, when i=j, $A_{ii}=0$, indicating that a robot’s pose does not form a relative pose constraint with itself. When i≠j, if there is an edge connecting vertices i and j, then $A_{ij}=A_{ji}=1$, meaning that a relative pose constraint exists between the poses of robot i and robot j in the multi-robot pose graph. Conversely, if there is no edge between vertices i and j, then $A_{ij}=A_{ji}=0$, indicating that there is no direct relative pose constraint between the poses of robot i and robot j in the current graph representation.

The definition of the Laplacian matrix L is closely related to the structure of the pose graph. Its elements $L_{ij}$ are defined based on the vertices and edges in the pose graph as follows:

(4) $$L_{ij}=\left\{\begin{array}{cc} \sum_{\left(i,j^{\prime }\right)\in \delta \left(i\right)}s_{e}, & i=j\\ -s_{e}, & \{i,j\}\in S\\ 0, & \{i,j\}\notin S \end{array}\right.$$

When i=j, the Laplacian matrix element is defined as $L_{ij}=\sum_{\left(i,j^{\prime }\right)\in \delta \left(i\right)}s_{e}$, where $\in \delta \left(i\right)$ represents the set of edges connected to vertex i. In the multi-robot pose graph, vertex i corresponds to a robot’s pose at a specific time. This equation indicates that the diagonal elements $L_{ij}$ of the Laplacian matrix are the sum of the weights of all edges connected to vertex i.

When {i,j}∈S, the Laplacian matrix element is defined as $L_{ij}=-s_{e}$, where {i,j}∈S indicates that there is an edge between vertices i and j, and $s_{e}$ represents the weight of this edge. In the multi-robot pose graph, edges represent relationships between robot poses. For example, if loop closure detection identifies an association between the poses of two robots at different locations, an edge is added between the corresponding vertices.

When {i,j}∉S, the Laplacian matrix element is defined as $L_{ij}=0$, indicating that there is no direct edge between vertices i and j. In the multi-robot pose graph, if loop closure is not detected between the poses of two robots, the corresponding element in the Laplacian matrix remains zero, signifying the absence of a direct connection.

Next, we determine the edge weights. For $e\in S^{\mathrm{local}}$, the weight $s_{e}$ is set to 1. This is because these edges represent fixed measurement constraints within a single robot, where the constraints between adjacent poses are relatively certain. Assigning a fixed weight to these edges is crucial for maintaining the fundamental structure and connectivity of the pose graph, helping to stabilize its foundational framework.

For $e\in S^{\mathrm{global}}$, the similarity between edges is computed using the Scan Context [28] LiDAR loop closure detection algorithm, and the bags of binary words for fast place recognition in image sequences (DBoW2) [29] visual loop closure detection algorithm. Equations (5) and (6), respectively, represent the similarity calculation formulas for LiDAR-based and vision-based loop closure detection:

(5) $$d\left(I^{q},I^{c}\right)=\frac{1}{N_{a}}\sum_{j=1}^{N_{a}}\left(1-\frac{c_{j}^{q}c_{j}^{c}}{\left\|c_{j}^{q}\right\|\left\|c_{j}^{c}\right\|}\right)$$

In this formula, $I^{q}$ and $I^{c}$ represent two different LiDAR scan frames, while $N_{a}$ denotes the number of angular bins used to partition the scan data for statistical analysis and computation. The feature value in the j-th angular bin of the query scan $I^{q}$ is represented by $c_{j}^{q}$, whereas $c_{j}^{c}$ corresponds to the feature value in the same angular bin of the reference scan $I^{c}$. The norms $\left\|c_{j}^{q}\right\|$ and $\left\|c_{j}^{c}\right\|$ are used for vector normalization. This formula quantifies the similarity between two scan frames by assessing the differences in their feature values across corresponding angular bins. If the comparison score is less than the set threshold ${d\left(I^{q},I^{c}\right)}_{th}$ (which is 0.1 in this paper), a loop closure is detected, and its similarity is recorded for subsequent weight calculation. If the comparison score exceeds the set threshold, it is treated as a false loop closure.

(6) $$s\left(v_{A}-v_{B}\right)=2\sum_{i=1}^{N}\left|v_{Ai}\right|+\left|v_{Bi}\right|-\left|v_{Ai}-v_{Bi}\right|$$

Here, $v_{A}$ and $v_{B}$ represent two different visual word vectors, while $v_{Ai}$ and $v_{Bi}$ denote the weight of the i-th visual word in image A and image B, respectively. The similarity between the two images is computed by comparing the differences in the intensity of their features. By finding the combination where the difference between the vector $v_{A}$ and $v_{B}$ is minimized, if the comparison score is less than the set threshold ${S\left(v_{A},v_{B}\right)}_{th}$ (0.25 in this paper), a loop closure is detected. The similarity is recorded for subsequent weight calculation, and if the comparison score exceeds the set threshold, it is treated as a false loop closure.

That is, the similarity obtained from LiDAR and visual loop closure is used to determine the edge weight $s_{e}=\left(d\left(I^{q},I^{c}\right),s\left(v_{A}-v_{B}\right)\right)$, where $s_{e}\in \left[0,\;1\right]$, indicating that the weight of candidate loop closure edges is determined based on the global matching similarity score, which falls within the range of $\left[0,\;1\right]$. For example, if the similarity score of the LiDAR or visual data scanned by two robots is 0.65, then $s_{e}$ is set to 0.65. This is because the reliability of candidate inter-robot loop closures largely depends on the degree of global matching similarity; the higher the similarity, the greater its potential value in constructing an accurate pose graph. By incorporating feature matching information into the pose graph structure evaluation, the system effectively enhances the accuracy of multi-robot localization and mapping.

Next, the enhanced pose graph Laplacian matrix is constructed:

(7) $$L\left(\omega \right)≜L^{S^{\mathrm{local}}}+\sum_{e\in S^{\mathrm{global}}}\omega_{e}L_{e}$$

where $\omega_{e}\in \left\{0,\;1\right\}$ is a binary variable used to determine the priority of candidate edge e. When $\omega_{e}=1$, it indicates that the candidate edge is selected for algebraic connectivity computation, meaning that the edge is considered to positively contribute to improving the overall quality of the pose graph. When $\omega_{e}=0$, the edge is excluded.

Finally, the algebraic connectivity of the pose graph is measured by computing the second smallest eigenvalue $\lambda_{2}$ of the rotationally weighted Laplacian matrix. The candidate subset is selected with the objective of maximizing $\lambda_{2}$:

(8) $$\begin{array}{c} \underset{\omega_{e}\in \{0,\;1\}}{max}\lambda_{2}(L(\omega ))\\ |\omega |=B \end{array}$$

Through the above-mentioned algebraic connectivity-based filtering method, compared to selecting candidate edges solely based on similarity, the overall structure of the pose graph is considered more comprehensively. This effectively avoids selecting loop closure edges that, despite having high similarity, do not substantially contribute to the overall pose graph optimization and may even lead to incorrect closures. This ensures that the selected candidate edges are more aligned with the actual loop closure situations.

#### 3.1.2. Local Matching Mechanism

Once the loop closure candidates are found, the system uses local features from sensors to accurately calculate the 3D relative position and orientation based on the type of sensor. Once the loop closure candidates are identified, the system uses local features obtained from sensors to calculate the 3D relative pose measurements based on the sensor type. To prevent redundant computations for the same loop closure and reduce communication overhead during geometric verification, a vertex coverage strategy is introduced. In situations where multiple robots are working together, if several loop closure candidates use the same vertex, only the necessary information about that shared vertex is sent, allowing for the quick calculations of all related position measurements.

Given n loop closure candidates with the corresponding vertex set $V=\left\{v_{1},v_{2},\cdots ,v_{m}\right\}$ and edge set $E=\left\{e_{1},e_{2},\cdots ,e_{k}\right\}$, where an edge $e_{i}=\left(v_{i1},v_{i2}\right)$ represents a loop closure relationship connecting two vertices. The binary variable $x_{v}$ is introduced, where $x_{v}=1$ indicates that vertex v is selected for transmission, and $x_{v}=0$ indicates that it is not transmitted. The objective is to minimize the number of selected vertices for transmission, which can be formulated as:

(9) $$min\sum_{v\in V}x_{v}$$

At the same time, the following constraint must be satisfied: for each edge $e_{i}=\left(v_{i1},v_{i2}\right)$, at least one of the vertices must be selected for transmission, i.e.,

(10) $$x_{v_{i1}}+x_{v_{i2}}\geq 1$$

By solving this integer programming problem, the optimal vertex transmission scheme can be obtained. Using algorithms [28,29] to compute relative pose measurements, the results of different candidate edges based on common vertices are compared. If the relative pose differences calculated from multiple candidate edges are too large, it indicates that there may be erroneous loop closure edges among them. Through this vertex covering strategy, during multi-robot collaborative exploration in complex environments, data transmission can be significantly reduced, while effectively verifying the correctness of candidate loop closure edges.

By solving this integer programming problem, the optimal vertex transmission scheme can be obtained. This vertex coverage strategy significantly reduces data transmission volume when multiple robots collaborate to explore complex environments.

At the same time, the segmented subgraphs often exhibit imbalances, leading to excessive workloads for some robots while others remain underutilized. To address this issue, the subgraphs need to be fine-tuned by adjusting their scale to ensure a balanced distribution of variables and computational complexity. This process reduces cross-connections and redundancy within subgraphs, ultimately improving computational efficiency.

Let the segmented subgraphs be $X_{1},X_{2},\ldots ,X_{N}$, with the number of nodes in each subgraph represented as $n_{1},n_{2},\ldots ,n_{N}$. The mean and variance of the number of nodes in the subgraphs are computed as follows:

(11) $$\bar{n}=\frac{1}{N}\sum_{i=1}^{N}n_{i}$$

(12) $$\sigma^{2}=\frac{1}{N}\sum_{i=1}^{N}(n_{i}-\bar{n}{)}^{2}$$

When balancing the subgraph partitioning, the criterion is the balance of the number of nodes and edges in each subgraph. Specifically, the variance $\sigma^{2}$ of the number of nodes in each subgraph should not exceed 5% of the total number of nodes across all subgraphs. By continuously adjusting the subgraph partitioning scheme, nodes are moved from subgraphs with a larger number of nodes to those with fewer nodes, ensuring that the size of each subgraph becomes more balanced.

#### 3.1.3. Multi-Level Graph Partitioning Loop Closure Detection Process

**Figure 2:**
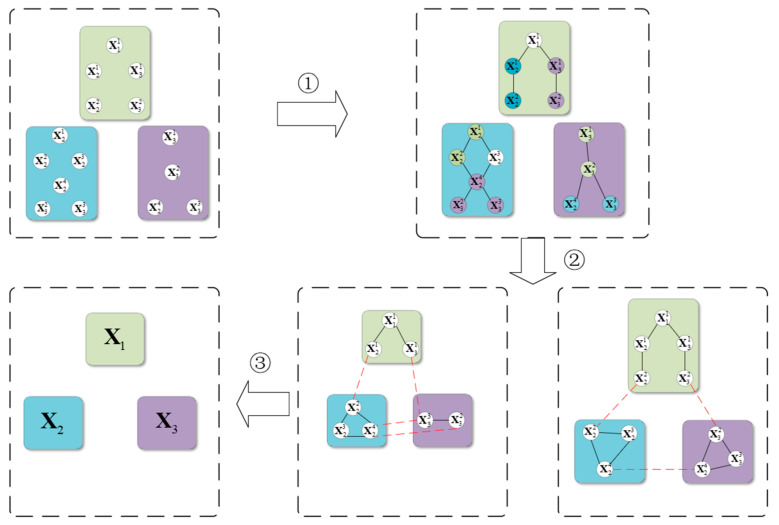
Subgraph partitioning.

When multiple robots work collaboratively, loop closure detection faces issues of high computational load and a high mismatch rate. Dividing the pose graph into multiple parts can reduce the scale and complexity of each subgraph, significantly decreasing the computational cost of calculating similarity scores and selecting candidate loop closure edges. With fewer nodes and edges within a subgraph, it becomes easier to perform precise local matching and verification, avoiding redundant measurements and reducing the possibility of mismatches. This section proposes a multi-level graph partitioning method aimed at dividing the original pose graph into multiple subgraphs suitable for distributed optimization in multi-robot systems. The specific steps are as follows:

As shown in Figure 2, the process begins with a node matching mechanism. In the first step, based on the evaluation function described in Section 3.1.1, highly coupled nodes are identified and matched, as represented by the black solid lines in the figure.

Next, in the second step, the edges and vertices obtained using the method in Section 3.1.2 are utilized for segmentation, partitioning the original graph into balanced subgraphs equal to the number of robots. Edge cutting is performed by removing certain edges to separate the graph into different parts, as indicated by the red dashed lines in the figure. Vertex cutting, on the other hand, removes specific vertices along with their associated edges to achieve graph segmentation. A balanced subgraph means that the number of nodes and edges in each subgraph is as evenly distributed as possible.

Finally, a proxy pose graph suitable for each robot is generated, ensuring that each subgraph is optimized for the corresponding robot’s pose graph computation. This guarantees that every robot can efficiently complete its assigned subgraph optimization task, ultimately achieving the goal of distributed pose graph optimization.

### 3.2. Improved Distributed Pose Graph Optimization Algorithm

Due to the challenges faced by classical distributed optimization algorithms, such as slow convergence and low optimization accuracy in complex environments with noise and large-scale multi-robot systems, this section proposes an improved approach. After performing front-end loop closure detection and subgraph segmentation, a distributed pose graph optimization algorithm is applied to the subgraphs. A robust cost function is integrated into the classical distributed optimization algorithm, allowing adaptive weight adjustments based on the size of the residuals, which effectively mitigates the influence of outliers. Furthermore, an improved iterative algorithm is introduced. By employing a subgraph block-based incremental update strategy, this approach can more quickly capture key local changes in the pose graph and rapidly adjust poses to minimize the objective function, thereby accelerating convergence speed. This provides more real-time pose estimation and map building results for multi-robot systems.

#### 3.2.1. Problem Description of the Improved Distributed Pose Graph Optimization

To enable collaborative multi-robot pose graph optimization in a distributed environment, it is essential to partition the constructed pose graph X appropriately. This section adopts the multi-level graph partitioning method proposed in the previous section to divide the pose graph X into N balanced subgraphs, thereby formulating the distributed pose graph optimization problem.

(13) $$X=\left\{X_{1},X_{2},\ldots ,X_{N}\right\}$$

Each variable in the pose subgraph $X_{i}$ is defined as follows, where the elements represent a set of keyframes that contain the most relevant information associated with robot i:

(14) $$X_{i}=\left(x_{i}^{\alpha },\cdots ,x_{j}^{\beta },\cdots ,x_{k}^{\gamma }\right)$$

Here, $x_{j}^{\beta }$ represents the pose of the j-th robot at the β-th keyframe. The subgraph $X_{i}$ contains the set of all local variables associated with the i-th robot. Each pose variable $x_{i}^{\alpha },x_{k}^{\gamma },x_{k}^{\gamma }\in SE(d)$, where ∀i,j,k∈[N] and $\forall \alpha ,\beta ,\gamma \in \left[\varepsilon_{i}\right]$. The term $\varepsilon_{i}$ denotes the set of all keyframe poses belonging to the i-th robot after the graph partitioning process.

Therefore, this section decomposes the global inter-robot relative measurement constraints into relative measurement constraints between robots within each subgraph block:

(15) $$\underset{{\left\{x_{i}\right\}}_{i=1}^{N}}{min}f\left(X\right)=\sum_{i=1}^{N}f_{i}\left(x_{i}\right)$$

(16) $$f_{i}\left(x_{i}\right)=\sum_{\left(x_{i}^{\alpha },x_{j}^{\beta }\right)\in E_{i}}w_{R}^{2}{∥R_{j}^{\beta }-R_{i}^{\beta }{\tilde{R}}_{i_{\alpha }}^{j_{\beta }}∥}_{F}^{2}+w_{T}^{2}{∥T_{j}^{\beta }-T_{i}^{\alpha }-R_{i}^{\alpha }{\tilde{T}}_{i_{\alpha }}^{j_{\beta }}∥}_{2}^{2}$$

In this formulation, $f_{i}\left(x_{i}\right)$ represents the objective function for the subproblem of relative measurement constraints between robots. The term ${∥R_{j}^{s}-R_{i}^{\tau }{\tilde{R}}_{i_{\tau }}^{j_{s}}∥}_{F}^{2}$ denotes the rotational error, where ${\tilde{R}}_{i_{\alpha }}^{j_{\beta }}$ represents the relative rotation measurement between robot i at keyframe α and robot j at keyframe β. Similarly, ${∥T_{j}^{\beta }-T_{i}^{\alpha }-R_{i}^{\alpha }{\tilde{T}}_{i_{\alpha }}^{j_{\beta }}∥}_{2}^{2}$ represents the translational error, where ${\tilde{T}}_{i_{\alpha }}^{j_{\beta }}$ denotes the relative translation measurement between robot i at keyframe α and robot j at keyframe β. The objective of this optimization problem is to determine the positions $t_{\alpha_{i}}$ and rotations $t_{\alpha_{i}}$ of robot i at different time instances. By minimizing this objective function, the discrepancy between the estimated poses of robot i and the actual measurements is minimized.

Therefore, the overall optimization problem for multi-robot back-end pose graph optimization can be formulated as:

(17) $$\underset{\begin{array}{c} X_{\alpha_{i}}\in SE(3)\\ \forall \alpha \in R,\forall i \end{array}}{min}\sum_{\alpha \begin{array}{c} \in \end{array}R}\sum_{i=1}^{n_{\alpha }-1}r_{\alpha_{i}}{\left(X_{\alpha_{i}},X_{\alpha_{i+1}}\right)}^{2}+\sum_{\left(\alpha_{i},\beta_{j}\right)\in L}\left(f_{i}\left(x_{i}\right)\right)$$

where $\sum_{\alpha \begin{array}{c} \in \end{array}R}\sum_{i=1}^{n_{\alpha }-1}r_{\alpha_{i}}{\left(X_{\alpha_{i}},X_{\alpha_{i+1}}\right)}^{2}$ represents the intra-robot optimization problem, Meanwhile, $\sum_{\left(\alpha_{i},\beta_{j}\right)\in L}\left(f_{i}\left(x_{i}\right)\right)$ corresponds to the subgraph optimization problem proposed in this section.

Moreover, since subgraph partitioning is based solely on similarity scores, perception aliasing may occur within subgraphs, leading to erroneous data associations and abnormal loop closures. This issue can introduce measurement errors in inter-robot constraints, as described in Equation (16), causing inaccurate relative pose estimates. Furthermore, incorrect loop closures may result in erroneous trajectory associations, significantly compromising the accuracy of trajectory estimation. To address this issue, this section incorporates a truncated least squares (TLS) robust cost function δ [30]. The function δ is applied to the loop closure residual $r_{\beta_{i}}^{\alpha_{i}}\left(X_{\alpha_{i}},X_{\beta_{j}}\right)$, where it adaptively adjusts the weight based on the magnitude of the residual. When the residual $r_{\beta_{i}}^{\alpha_{i}}\left(X_{\alpha_{i}},X_{\beta_{j}}\right)$ is large, indicating that the measurement is affected by noise or contains outliers, the function δ reduces the corresponding weight to mitigate the impact of outliers on the overall optimization. Conversely, when the residual is small, the weight remains unchanged or is only slightly adjusted, ensuring that valid measurements contribute effectively to the optimization. Consequently, Equation (17) is reformulated as follows:

(18) $$\underset{\begin{array}{c} X_{\alpha_{i}}\in SE(3)\\ \forall \alpha \in R,\forall i \end{array}}{min}\sum_{\alpha \begin{array}{c} \in \end{array}R}\sum_{i=1}^{n_{\alpha }-1}r_{\alpha_{i}}{\left(X_{\alpha_{i}},X_{\alpha_{i+1}}\right)}^{2}+\sum_{\left(\alpha_{i},\beta_{j}\right)\in L}\delta \left(f_{i}\left(x_{i}\right)\right)$$

The weight update for each residual function using the TLS robust cost function is given by:

(19) $$\delta \leftarrow \left\{\begin{array}{cc} 0, & if\;{f_{i}\left(x_{i}\right)}^{2}\in \left[\frac{\mu +1}{\mu }{\bar{c}}^{2},+\infty \right]\\ \frac{\bar{c}}{f_{i}\left(x_{i}\right)}\sqrt{\mu (\mu +1)}-\mu , & if\;{f_{i}\left(x_{i}\right)}^{2}\in \left[\frac{\mu }{\mu +1}{\bar{c}}^{2},\frac{\mu +1}{\mu }{\bar{c}}^{2}\right]\\ 1, & if\;{f_{i}\left(x_{i}\right)}^{2}\in \left[0,\frac{\mu }{\mu +1}{\bar{c}}^{2}\right] \end{array}\right.$$

In this method, the weight coefficient depends solely on the current residual error $f_{i}\left(x_{i}\right)$, control parameter μ, and the threshold $\bar{c}$ for the TLS (total least squares) cost. The control parameter μ is used to adjust the sensitivity of residual weight changes, and μ and $\bar{c}$ work together. The threshold $\bar{c}$ directly determines the truncation range for the residual. When the residual $f_{i}\left(x_{i}\right)$ exceeds $\bar{c}$, the value of the cost function is truncated to $\bar{c}$, effectively limiting the impact of outliers on the overall optimization result. For a detailed definition, please refer to [30]. The specific values of *b*b and *c*c are determined based on the experimental environment. When the residual ${f_{i}\left(x_{i}\right)}^{2}\in \left[\frac{\mu +1}{\mu }{\bar{c}}^{2},+\infty \right]$, the weight δ is set to 0, indicating that the measurement is classified as an outlier and is ignored in subsequent optimization steps to prevent its interference with trajectory estimation. When the residual ${f_{i}\left(x_{i}\right)}^{2}\in \left[\frac{\mu }{\mu +1}{\bar{c}}^{2},\frac{\mu +1}{\mu }{\bar{c}}^{2}\right]$, the weight δ is computed using the formula $\frac{\bar{c}}{f_{i}\left(x_{i}\right)}\sqrt{\mu (\mu +1)}-\mu$ This transitional handling gradually reduces the weight of measurements approaching the outlier threshold, preventing abrupt weight changes that could negatively impact the optimization process. When the residual ${f_{i}\left(x_{i}\right)}^{2}\in \left[0,\frac{\mu }{\mu +1}{\bar{c}}^{2}\right]$, the weight δ is set to 1, indicating that the measurement is considered reliable and retains its original influence in the optimization.

#### 3.2.2. Improved Iterative Optimization Algorithm

Due to the nonlinear and manifold structure of pose variables, traditional Euclidean space optimization methods are not applicable. Therefore, the Riemannian Block Coordinate Descent (RBCD) solver [19] is employed for distributed optimization. The RBCD algorithm performs block coordinate descent on a Riemannian manifold, leveraging the properties of Riemannian geometry to handle the nonlinearity and constraints of pose variables. By iteratively updating robot poses, the algorithm minimizes the objective function, ultimately yielding more accurate trajectory estimates.

In the field of distributed pose graph optimization, efficient and precise algorithms are crucial for achieving accurate collaborative localization and mapping in multi-robot systems. While the Riemannian Block Coordinate Descent (RBCD) algorithm partially addresses distributed pose graph optimization and ensures convergence, it performs optimization on the entire pose graph, resulting in excessive computational overhead for each robot and reducing the algorithm efficiency. Consequently, it suffers from slow convergence and limited solution accuracy. To address these limitations, this section proposes an improved RBCD-based distributed pose graph optimization algorithm. The global pose graph optimization is decomposed into distributed subgraph optimizations, where each robot α∈R is responsible for estimating its own trajectory $X_{\alpha }≜\left\{X_{\alpha_{i}},i=1,\ldots ,n_{\alpha }\right\}$. During execution, the proposed iterative optimization is performed in blocks using the subgraph partitioning strategy introduced in Section 3.1. Each subgraph $X_{i}$ is iteratively optimized by leveraging internal pose transformations and relative pose constraints provided by loop closure frames associated with other robots. This approach reduces the communication overhead related to unnecessary pose exchanges while maintaining convergence, thereby improving the overall efficiency of the algorithm.

**Figure 3:**
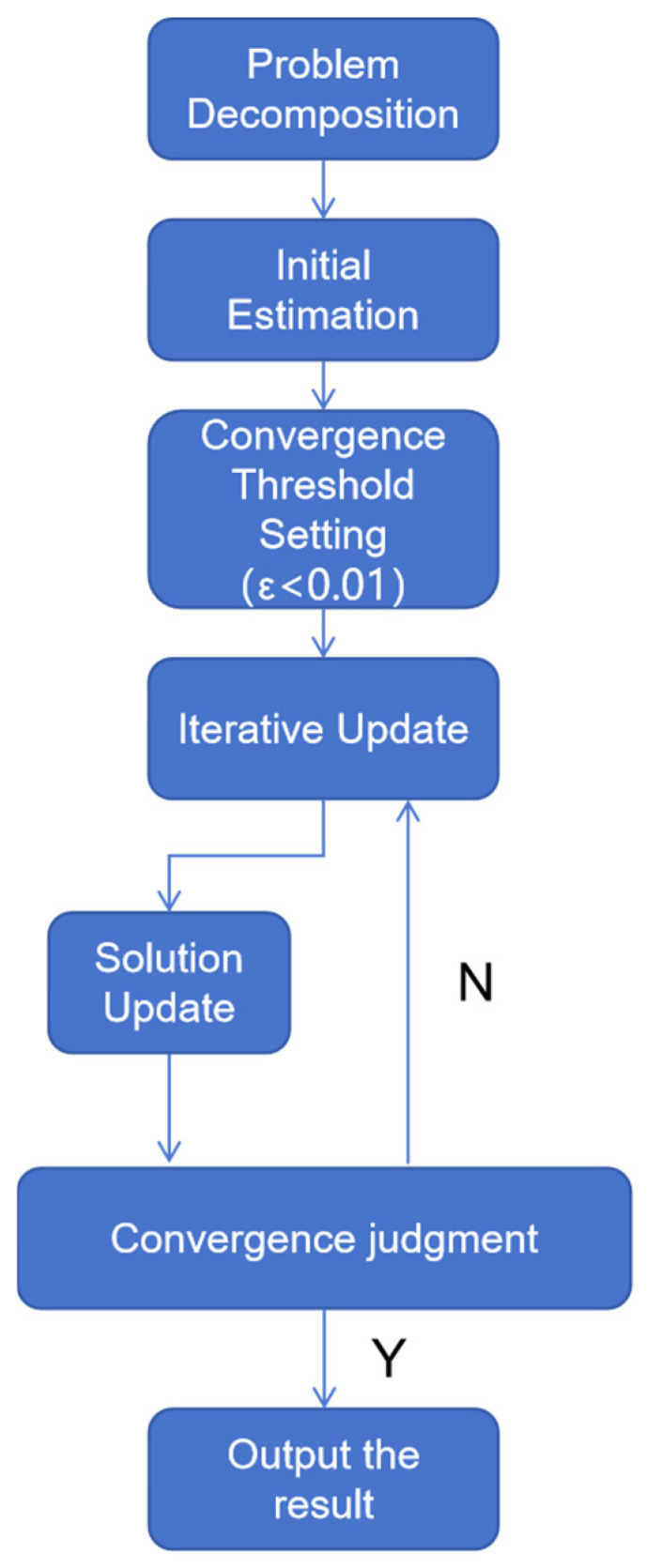
Flowchart of the improved RBCD algorithm.

The complete steps of the improved RBCD algorithm are given as follows, with the specific process shown in Figure 3:

1. Problem decomposition: The trajectory optimization problem for the entire multi-robot system is partitioned into N submodules using the subgraph segmentation method designed in Section 3.1. Each submodule corresponds to the trajectory optimization of a single robot and its associated robots. Within the subgraph segmentation, each submodule only needs to process the local objective function $f_{i}\left(x_{i}\right)$:
  (20) $${minf}_{X_{i}\in X}\left(X_{i}\right)≜f\left(X_{i},{\hat{X}}_{\left[N]\setminus \{i\right\}}\right)$$
  Here, $X_{i}$ represents the variable block corresponding to the selected robot, while ${\hat{X}}_{\left[N]\setminus \{i\right\}}$ denotes the remaining variable blocks that are simultaneously updated during the iteration of $X_{i}$. This transformation converts the originally complex multi-variable optimization problem into a set of relatively simpler subproblems that are concurrently optimized for different variable blocks.
2. Initial estimation and convergence threshold setting: Each robot within its respective submodule performs an initial estimation of its pose using a localization algorithm. These initial estimates collectively form the initial solution $Z^{0}$. In this study, a convergence threshold of ϵ<0.01 is chosen as the criterion for determining convergence.
3. Iterative update: For each variable block $X_{i}$, the variable values within its subgraph are updated by minimizing the objective function $f_{X_{i}\in X}\left(X_{i}\right)$. This process iteratively refines $Z^{0}$, reducing the error between the estimated and actual relative poses of the robots. The optimization problem at this stage can be expressed as:
  (21) $$Z_{X_{i}}^{k+1}=\underset{Z_{X_{i}}\in M_{X_{i}}}{\arg min}\;f\left(Z_{-i_{k}}^{k},Z_{i_{k}}\right)$$
  where $Z_{-i_{k}}^{k}$ represents the values of all variables except $X_{i}$ at the k-th iteration, and $M_{X_{i}}$ denotes the Riemannian submanifold corresponding to the coordinate block $X_{i}$, which defines the search space for $Z_{i_{k}}$.
4. Solution update: Combine the updated results of all coordinate blocks to obtain the solution $Z^{k+1}$ for the k+1-th iteration.
5. Convergence criterion: Compute the Riemannian gradient norm $\left\|\nabla_{M}f\left(Z^{k+1}\right)\right\|$ at $Z^{k+1}$. The Riemannian gradient $\nabla_{M}f\left(Z^{k+1}\right)$ represents the derivative of the objective function on the Riemannian manifold M, indicating the rate of change of the function value along the manifold. Compare the computed gradient norm with the predefined convergence threshold ϵ. If $\left\|\nabla_{M}f\left(Z^{k+1}\right)\right\|\leq \epsilon$, the algorithm is considered to have converged, and the iteration terminates. Otherwise, set k=k+1 and return to the iteration update step for the next optimization cycle.
6. Output result: Upon the convergence of the algorithm, the optimal solution $Z^{*}$ is obtained, which minimizes the objective function within the given error tolerance. The final output $Z^{*}$ represents the optimal pose estimation for the robots.

## 4. Experimental Validation

To validate the effectiveness of the proposed algorithm, experiments were conducted using both the public KITTI dataset and a custom-built dataset. The KITTI00 sequence represents urban streets with abundant traffic signs and infrastructure, where lighting conditions are significantly affected by building shadows, and scenes contain rich texture details. The KITTI02 sequence includes road gradient variations, notable lighting changes, and disturbances from dynamic vehicles and pedestrians. The KITTI05 sequence features complex intersections, diverse building types, and varied terrain, posing significant challenges for algorithm testing. The custom dataset was collected through vehicle-mounted experiments conducted in a campus environment. This campus setting is relatively structured, featuring distinctive elements such as buildings, roads, and vegetation, along with dynamic interference from moving pedestrians and vehicles. In the KITTI dataset experiments, three robots were configured to collaboratively perform verification. Each robot was equipped with a high-precision LiDAR and a visual camera. The LiDAR provides accurate distance measurements, while the camera captures rich texture features, making the platform suitable for perception in complex environments. This section evaluates the proposed algorithm’s performance through five experiments: algebraic connectivity analysis, loop closure precision–recall metrics, the time consumption of loop detection algorithms, comparisons of convergence speed and optimization accuracy, and the trajectory comparisons between the proposed and other algorithms against ground truth. Table 2 lists the detailed parameters of each sequence used in the experiments. The simulation environment configuration used in this paper is shown in Table 3.

### 4.1. Algebraic Connectivity Experiment

**Figure 4:**
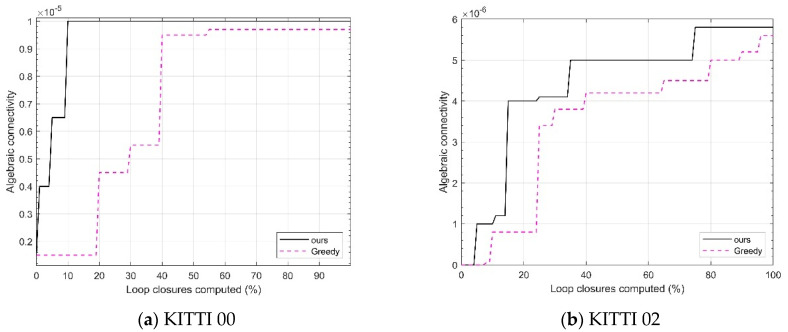
Algebraic connectivity comparison on KITTI sequences.

First, an algebraic connectivity experiment is conducted, which is a key metric for evaluating the performance of the pose graph. The value of algebraic connectivity reflects the degree of connectivity between nodes in the pose graph. In other words, it measures the connectivity between loop closures. A higher algebraic connectivity indicates smoother information flow between nodes, which results in higher accuracy for robot pose estimation and map construction based on the pose graph. This paper compares the algebraic connectivity of the proposed algorithm with the greedy algorithm used in reference [22]. By doing so, it is possible to intuitively see which method better optimizes the pose graph structure and enhances the overall system performance. Figure 4 shows the results of running the KITTI00 and KITTI02 sequences, gradually processing loop candidates according to the calculated loop closure percentage. For each loop closure percentage node, algebraic connectivity is recorded under both the proposed algorithm and the greedy algorithm, and the corresponding curves are plotted.

As shown in Figure 4, for both sequences, as the calculated loop closure percentage increases, the algebraic connectivity value corresponding to the algorithm in this chapter is generally higher than that of the Greedy algorithm. This indicates that the proposed algorithm in this chapter is better at handling the priority of candidate loop closures and optimizing the pose graph structure, resulting in tighter connections between the nodes in the pose graph and more effective information transmission, thus improving the estimation accuracy.

### 4.2. Precision and Recall Experiment

This section evaluates the loop closure detection performance using precision–recall (PR) curves, where the *x* axis represents recall and the *y* axis represents precision. The better the loop closure detection algorithm, the more the PR curve tends toward the upper right corner, indicating that the algorithm can maintain high precision even with high recall.

The proposed algorithm is compared with three other loop closure detection algorithms: ISC [10], M2DP [8], and ESF [9]. Table 4 presents the $F_{1}$ scores of different algorithms on the KITTI dataset. From the table, it can be observed that the $F_{1}$ scores of the proposed algorithm outperform other loop closure detection algorithms in most KITTI sequences. For example, in the KITTI00 sequence, the $F_{1}$ score of the proposed algorithm reaches 0.9854, which is approximately 13.5% higher than that of the ISC algorithm. This indicates that the proposed algorithm can accurately identify loop closures in multi-robot collaborative operations and is more robust to rotational variations.

**Figure 5:**
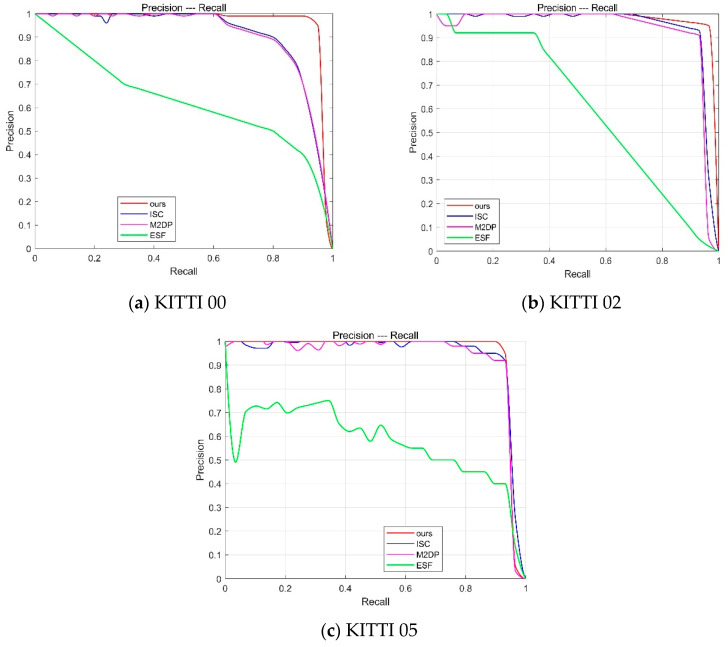
PR curve for the KITTI dataset.

This section qualitatively evaluates the loop closure detection performance of the proposed algorithm based on the PR curve. In the experiments conducted on the KITTI dataset, the performance of the loop closure detection schemes is measured using the PR curves shown in Figure 5. The curves in different colors represent different loop closure detection algorithms. The experimental results show that, compared to other algorithms, the proposed algorithm demonstrates a superior detection performance on the KITTI dataset. The higher recall rate indicates an effective reduction in drift error, while the higher precision ensures that false loop closures are not incorporated into the map. The ESF algorithm performs poorly on the KITTI sequences due to its heavy reliance on histograms, making it difficult to accurately distinguish between the different locations in environments with minimal variation. This leads to loop closure detection failure. The M2DP algorithm exhibits excellent precision at low recall rates because it accurately detects forward loop closures. However, it does not account for rotational and translational changes, causing reverse loop closures to be overlooked. The experimental results show a sharp decline in the curve slope. The ISC algorithm performs relatively well but lacks theoretical rotational invariance. This method relies on brute-force matching and vertical structural information, making its detection performance limited when vertical height changes are small in the environment. In conclusion, the loop closure detection scheme proposed in this chapter shows promising performance on the KITTI dataset and is beneficial for improving the localization accuracy of SLAM systems.

### 4.3. Comparison of Computational Time for Loop Closure Detection Algorithms

**Figure 6:**
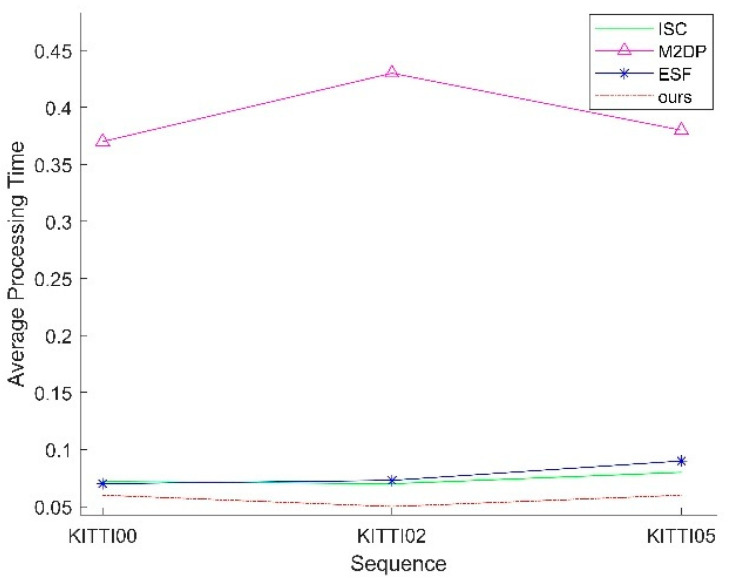
Average processing time on the KITTI dataset.

To validate the efficiency of the proposed loop closure detection algorithm, this section calculates and accumulates the processing time required for each frame, from projection to identifying the most similar candidate frame. The total processing time is then divided by the total number of frames to obtain the average processing time per frame. Figure 6 presents the average processing time per frame for different algorithms on the KITTI dataset. It can be observed that the proposed loop closure detection algorithm achieves a lower average processing time per frame compared to other algorithms.

### 4.4. Comparison of Iteration Speed and Solution Accuracy Experiments

To verify the effectiveness of the proposed subgraph-based distributed pose graph optimization method in this paper, the KITTI dataset, which includes the simulated scenarios mentioned earlier, is used. These simulated datasets allow for precise control over data characteristics and scale, facilitating targeted algorithm testing. For the improved iterative algorithm, key prior parameters are set: ϵ=0.01, which serves as the termination criterion.

**Figure 7:**
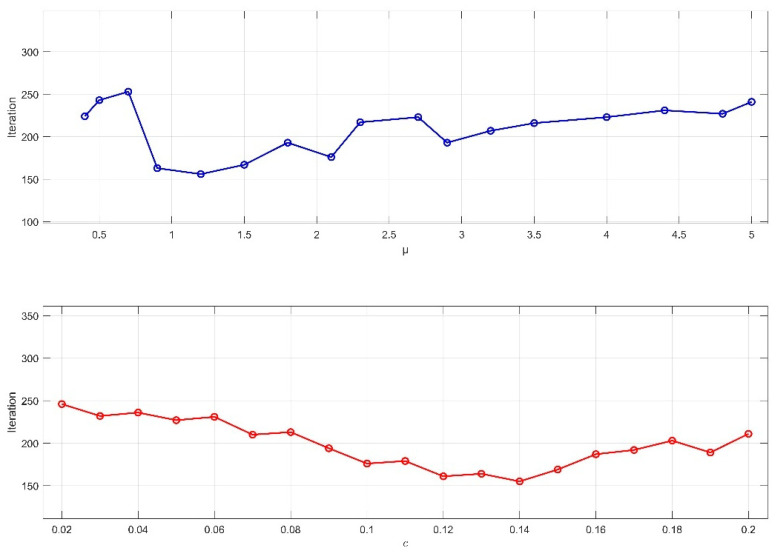
Effect of parameter values μ and $\bar{c}$ on the number of iterations in the KITTI00 sequence.

**Figure 8:**
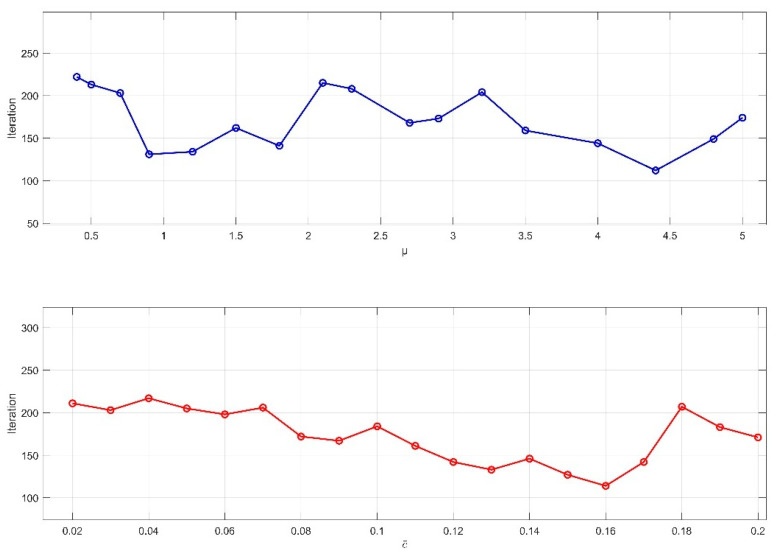
Effect of parameter values μ and $\bar{c}$ on the number of iterations in the KITTI02 sequence.

First, the values of the control parameter μ and the TLS cost threshold $\bar{c}$ are determined based on the characteristics of the dataset environment. As shown in Figure 7 and Figure 8, for the KITTI00 and KITTI02 sequences, different combinations of μ and $\bar{c}$ are used to record the number of iterations required to meet the termination condition. The results indicate that, in the urban road scenario of sequence 00, where lighting is heavily affected by building occlusion and scene textures are complex, setting μ = 1.2 and $\bar{c}$ = 0.14 leads to fewer iterations. In contrast, in sequence 02, which includes varying road slopes, significant lighting changes, and dynamic interferences from vehicles and pedestrians, setting μ = 4.4 and $\bar{c}$ = 0.16 achieves convergence in fewer iterations.

Secondly, a comparative experiment is conducted between the improved iterative algorithm proposed in this paper and the RBCD algorithm. Both algorithms are independently executed on the dataset, and various key metrics during execution, such as the number of iterations and gradient norm variations, are recorded in detail.

**Figure 9:**
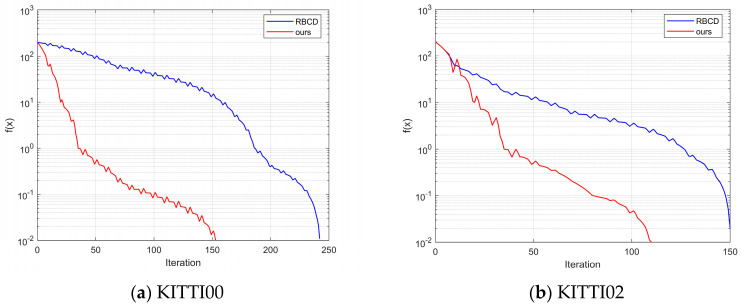
Iterative comparison experiment on the dataset.

**Figure 10:**
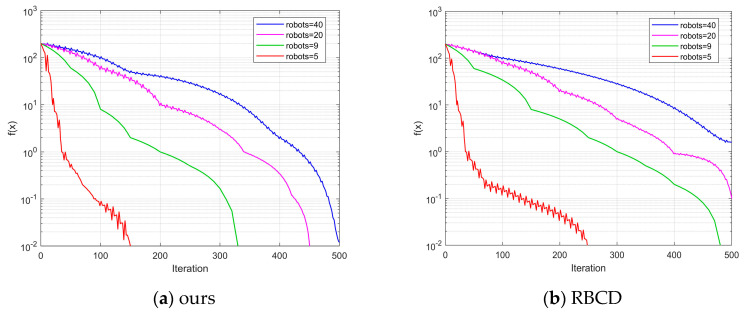
Impact of different robot quantities on algorithm performance in sequence 00.

As shown in Figure 9, the back-end optimization process involving three robots is presented for the KITTI dataset. The horizontal axis represents the number of iterations, while the vertical axis indicates the difference between the objective function value and the optimal value. Experimental results reveal that the proposed improved iterative algorithm demonstrates a significant advantage in terms of the number of iterations required to converge to the optimal solution. In the KITTI00 and KITTI02 sequences, the proposed algorithm satisfies the termination criterion in fewer than 150 iterations, whereas the RBCD algorithm requires more iterations. This strongly validates the substantial improvement in convergence speed achieved by the proposed method, enabling faster pose graph optimization and enhancing the real-time performance of multi-robot systems. Figure 10 illustrates the impact of different numbers of robots on algorithm performance. As the iterations progress, the difference between the objective function value and the optimal value gradually decreases for various robot counts (5, 9, 20, 40). This demonstrates that the proposed algorithm maintains a fast iteration speed and achieves convergence across different robot configurations. In contrast, the RBCD algorithm exhibits divergence in scenarios involving a larger number of robots. Compared to the RBCD algorithm, the proposed method consistently converges with fewer iterations, maintaining both high convergence efficiency and stable solution accuracy, even in large-scale multi-robot experiments.

To further verify the operational efficiency of the improved iterative algorithm proposed in this paper, the running times of both the proposed algorithm and the RBCD algorithm were recorded on sequences 00 and 02. Table 5 presents the average execution time over 10 runs for each algorithm under different numbers of robots (3, 5, 9, 20, 40). As shown in Table 5, the proposed algorithm consistently exhibits shorter execution times on both sequences, demonstrating higher computational efficiency compared to the RBCD algorithm.

### 4.5. Positioning Accuracy Comparison Experiment

To validate the feasibility and effectiveness of the proposed multi-robot cooperative SLAM system in outdoor environments, this study constructs a mobile robot system and designs a modular sensor data acquisition platform that can be deployed on mobile robots, enabling efficient collection and processing of various types of data. Based on this experimental system, the positioning accuracy of multi-robot SLAM is empirically validated and demonstrated using both simulation datasets and a custom dataset. The Absolute Trajectory Error (ATE) is employed to evaluate algorithm performance, which quantifies the overall trajectory deviation as the mean Euclidean distance between corresponding points on the estimated and ground truth trajectories.

#### 4.5.1. Simulation Dataset Comparison Experiment

To evaluate the accuracy of the proposed multi-robot SLAM system, the dataset was divided into three sub-sequences, ensuring overlapping regions between consecutive sub-sequences. The KITTI dataset was partitioned into three timestamp-synchronized subsets, with timestamps adjusted to include overlapping portions. The proposed method was compared with Distributed scan context-enabled multi-robot lidar slam with two-stage global-local graph optimization (DiSCo-SLAM) [14] and a distributed collaborative LiDAR SLAM framework for a robotic swarm (DCL-SLAM) [27]. DiSCo-SLAM is a recently introduced open source multi-robot SLAM system based on LiDAR sensors, utilizing scan context descriptors for inter-robot loop closure detection. DCL-SLAM is an open source, fully distributed collaborative LiDAR SLAM framework based on ROS, employing lightweight LiDAR-Iris descriptors and point-to-point communication. It integrates interchangeable front-end odometry, distributed loop closure detection, and back-end optimization modules, offering high accuracy and low bandwidth consumption. DCL-SLAM is designed to accommodate various LiDAR configurations, robotic platforms, and complex environments. A comparative experiment was conducted to validate the localization accuracy of the proposed algorithm against existing methods. The KITTI dataset sequences 05 and 08 were selected as test cases for this evaluation.

Table 6 presents detailed information on the selected KITTI dataset sequences used in this study, including the environment classification of sequences 05 and 08, as well as the trajectory lengths of different robots in each sequence. The trajectory length reflects the movement range of the robots within the corresponding environment and the amount of collected data. Longer trajectories indicate broader scene coverage and greater data variation, providing a more rigorous evaluation of the SLAM system’s stability and accuracy over extended operation periods. In the KITTI dataset experiments, the simulated network latency ranged from 20 to 100 ms, and the packet loss rate was set between 5% and 15%.

**Figure 11:**
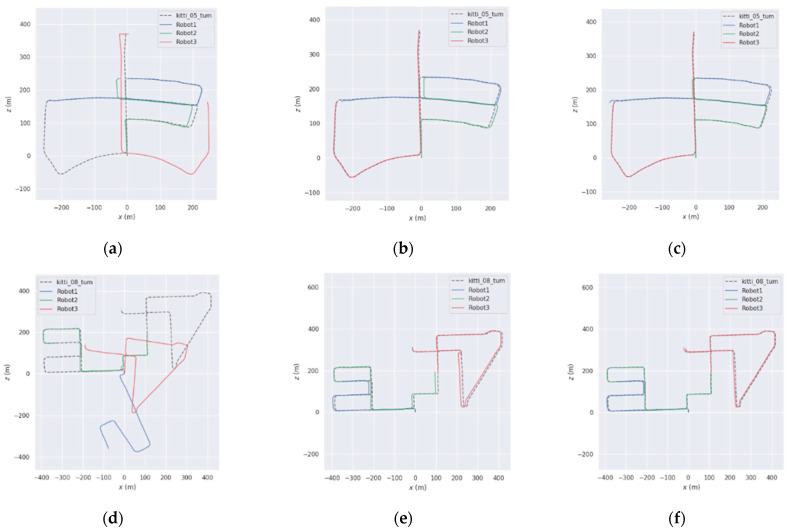
Trajectory results on KITTI sequences ((**a**–**c**) for sequence 05, (**d**–**f**) for sequence 08), (**a**) DiSCo-SLAM; (**b**) DCL-SLAM; (**c**) ours; (**d**) DiSCo-SLAM; (**e**) DCL-SLAM; (**f**) ours.

As shown in the figure, Figure 11a–f presents the trajectory results of different methods on sequences 05 and 08. It is important to note that the trajectories in multi-robot SLAM are aligned with the ground truth. Therefore, any trajectory that fails to merge with others is considered a failure. As listed in Table 7, when DiSCo-SLAM fails due to the absence of inter-robot loop closures, the proposed method and DCL-SLAM successfully merge all submaps in sequences 05 and 08. In the successfully mapped sequences 05 and 08, the proposed method achieves a lower absolute trajectory error (ATE) compared to both DCL-SLAM and DiSCo-SLAM. Specifically, the proposed method improves localization accuracy by approximately 6.53% and 52.77% over DCL-SLAM on sequences 05 and 08, respectively. These results indicate that the proposed approach outperforms the other two methods in both sequences, achieving the best performance. These results indicate that the proposed method achieves higher accuracy in the segmented public KITTI odometry sequences.

#### 4.5.2. Custom Dataset Comparison Experiment

**Figure 12:**
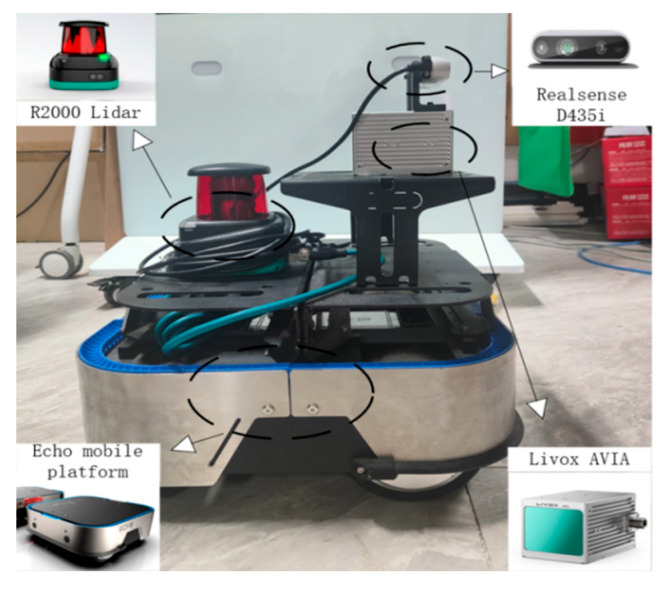
Mobile robot data collection system.

To verify the practicality of the proposed multi-robot SLAM algorithm in real-world scenarios, this section presents a vehicle-mounted experiment conducted on a university campus. During the experiment, a robot was controlled to follow a predefined trajectory while collecting data in a large-scale outdoor environment. Both single-robot and multi-robot SLAM tests were performed. The coordinate data collected using an R2000 LiDAR, which employs a retroreflector-based trilateration method with an accuracy of ±3 mm [31], were used as the ground truth reference for this experiment. The accuracy of the proposed algorithm was evaluated using the absolute trajectory error (ATE). Figure 12 presents the custom-built mobile robot data collection platform, which consists of an Echo mobile platform (Qiyuan Robotics Co. Ltd., Dongguan, China), a Livox AVIA multi-line LiDAR (DJI Technology Co. Ltd., Shenzhen, China), a Realsense D435i camera (Intel Corporation, Santa Clara, CA, USA), an IMU integrated into the camera, and an R2000 LiDAR (Pepperl+Fuchs GmbH, Mannheim, Germany). The Livox AVIA multi-line LiDAR features high resolution and a large field of view, allowing for the rapid acquisition of point cloud data over large areas. Its measurement accuracy reaches ±2 cm, providing precise geometric information for map building. The Realsense D435i camera not only captures high-quality color images but also acquires depth information. Its color image resolution is 1920 × 1080, while its depth image resolution is 1280 × 720, with a frame rate of up to 30 fps, offering rich data for visual feature extraction and loop closure detection. The camera’s built-in IMU can measure the robot’s acceleration and angular velocity in real time, providing inertial information for pose estimation, thereby enhancing the accuracy and stability of localization. The specifications of these sensors have been detailed in the previous section. The computational platform used in the experiment was an Acer (Acer Inc., Taipei, Taiwan Province, China) laptop equipped with a 12th Gen Intel^®^ Core™ i5-12500H CPU and 16 GB of RAM. The software environment was based on Ubuntu 18.04 with the ROS Melodic framework. In this section, the network delay is set to 30–80 ms, and the packet loss rate is set to 8–12%.

**Figure 13:**
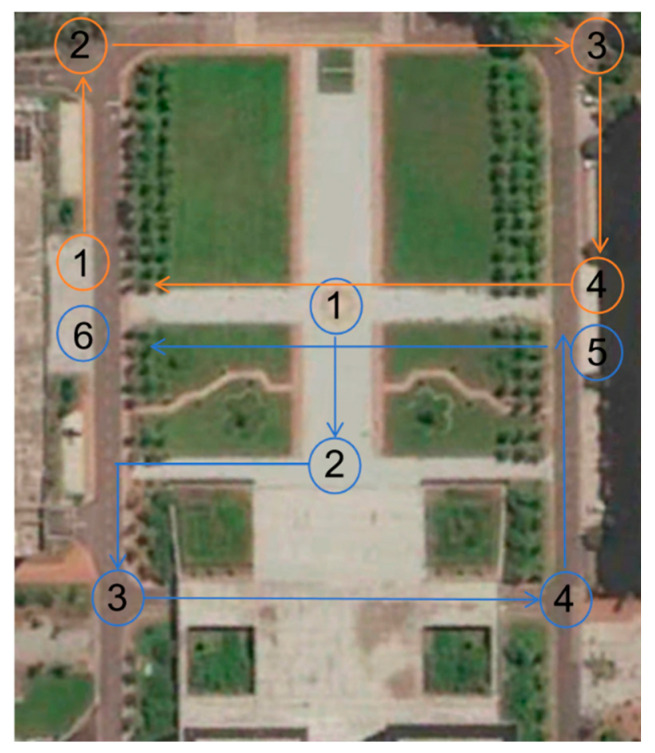
Planned trajectories of each robot.

**Figure 14:**
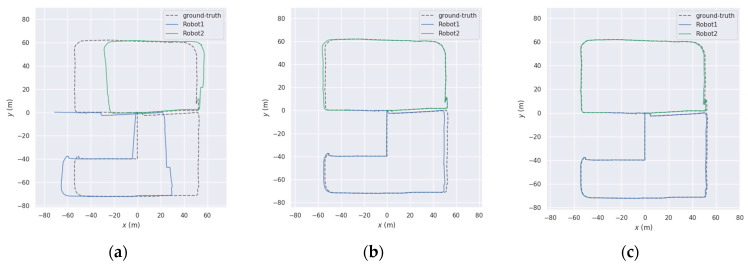
Trajectory results of the custom test dataset: (**a**) DiSCo-SLAM; (**b**) DCL-SLAM; and (**c**) ours.

To validate the proposed multi-robot SLAM system in real-world environments, considering the limitations of sensor equipment, data collection for the multi-robot SLAM system was conducted using a single robot. The recorded data were then processed using ROS’s data recording and playback functionality, similar to the verification approach used for public datasets. The recorded dataset was divided into two sub-sequences, which were simultaneously replayed on a single device to simulate the effect of multiple robots operating concurrently. In this experiment, sensor data from the robot’s operation were collected over an area of approximately 13,000 square meters to validate the effectiveness of the proposed algorithm. To evaluate the performance of the proposed multi-robot SLAM algorithm, Table 8 presents the trajectory length information for each robot in the custom test dataset. A comparative analysis of localization trajectories between DiSCo-SLAM, DCL-SLAM, and the proposed algorithm was conducted within the custom test dataset. Figure 13 illustrates the planned trajectories of two robots, the trajectory of Robot 1 is the blue track in the figure, and the operation sequence is 1-2-3-4-5-6. The trajectory of Robot 2 is the orange track in the figure, and the operation sequence is 1-2-3-4-1, while Figure 14 presents the localization trajectory evaluation results for different algorithms. As shown in the figure, the proposed algorithm successfully completes high-quality mapping in large-scale outdoor environments, maintaining a trajectory that closely aligns with the reference trajectory with effective loop closure. The ATE results are presented in Table 9, where it is observed that DiSCo-SLAM fails due to the absence of loop closure. Compared to DCL-SLAM, the proposed algorithm achieves a 32.8% improvement in localization accuracy.

**Figure 15:**
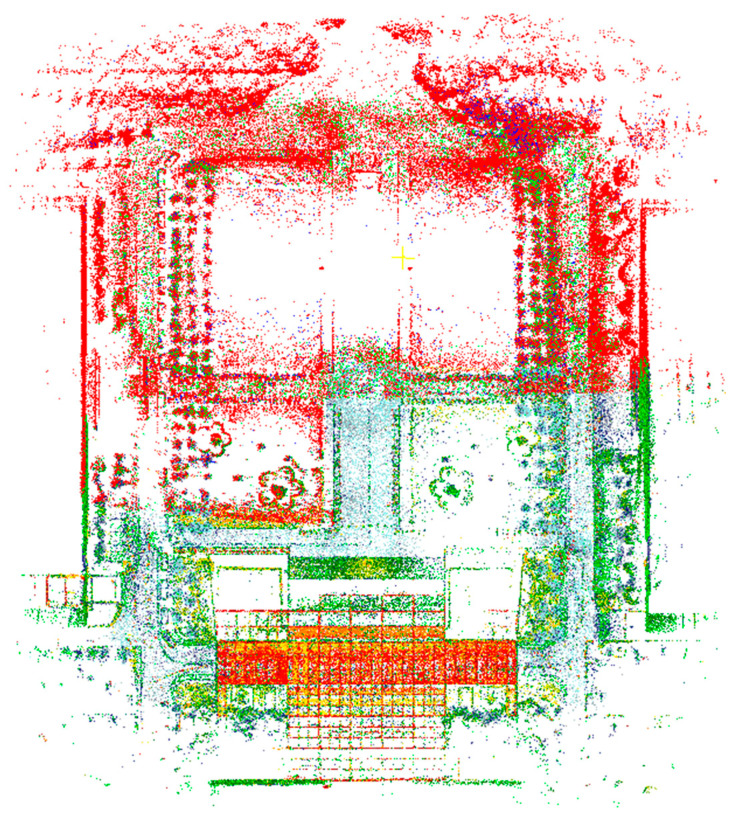
Global map of the environment constructed by two robots while running on the campus dataset.

Exception: This section presents the global map constructed by the proposed algorithm while operating with two robots. Different colors are used to distinguish point cloud data collected by different robots, representing the global maps built by Robot 1 and Robot 2 during algorithm execution, displayed in the global coordinate system (see Figure 15).

## 5. Conclusions

This paper presents several optimizations for multi-robot cooperative SLAM algorithms. To address the inefficiency and high computational cost of front-end loop closure detection in multi-robot systems, particularly under communication and computational constraints in cooperative settings, this paper proposes a communication-constrained loop closure detection algorithm based on algebraic connectivity maximization. By selecting effective loop closure candidates and avoiding redundant measurements, the proposed method reduces communication and computational overhead, thereby improving localization accuracy and autonomy in multi-robot cooperative SLAM. Experimental results on the KITTI dataset demonstrate the superiority of the proposed algorithm, significantly reducing the per-frame processing time. On the KITTI02 dataset, the proposed method achieves an $F_{1}$ score of 0.8961, improving performance by approximately 8.5–51.5% compared to the ISC, M2DP, and ESF algorithms.

To enhance the accuracy and convergence speed of pose optimization in multi-robot cooperative SLAM, this paper introduces an improved distributed robust pose graph optimization algorithm. By integrating a subgraph-based distributed pose graph optimization model with a robust cost function for outlier rejection, the proposed method enhances computational efficiency and optimization accuracy. Experimental results indicate that the proposed approach significantly outperforms traditional methods in both convergence speed and optimization accuracy.

Regarding localization accuracy, the proposed algorithm improves performance on the KITTI dataset by approximately 6.53% and 52.77% compared to DCL-SLAM. In the self-collected dataset, it achieves a 32.8% improvement over DCL-SLAM. These results demonstrate the effectiveness of the proposed method in large-scale outdoor environments, highlighting its feasibility and advantages in real-world applications.

Although this paper reduces node data transmission through multi-level graph partitioning, it does not explore the impact of communication network topology on the algorithm’s performance in depth. Future work will include experiments in different complex scenarios, such as testing the algorithm in environments with large dynamic obstacles, systems with different types of sensors, and robots with various characteristics, to evaluate the adaptability and robustness of the algorithm. Furthermore, robustness assessment in complex scenarios still need to be expanded. In dynamic environments, moving object interference affects data extraction, and future tests will be conducted in scenes like squares and intersections to simulate high-noise scenarios, analyze the influencing patterns, and improving data processing or optimization algorithms to enhance noise resistance. Studying these complex scenarios will provide support for algorithm development and help promote its widespread application.

## Figures and Tables

**Table 1:** Advantages and disadvantages of traditional methods.

Algorithm	Advantages	Disadvantages
COVINS-G [15]	Multi-camera algorithms estimate loop closure constraints based on 2D image processing.	Loop closure fails in scenarios with severe occlusion or sparse feature points.
DDF-SAM [16]	Decentralized data fusion is achieved using the extended smoothing and mapping method.	In environments with high latency and packet loss, module data transmission synchronization is hindered.
RBCD [19]	It can obtain the global optimal solution, is distributed and efficient, and ensures privacy.	The iteration speed is slow, and it is difficult to handle outlier measurements.
DiSCo-SLAM [14]	Data-efficient and stable localization	Relies on feature matching, resulting in lower optimization accuracy.
DCL-SLAM [27]	High accuracy, low bandwidth.	Relies on single robot odometry accuracy, with limited resistance to interference.

**Table 2:** Detailed parameters of the KITTI dataset.

Sequence	Number of Frames	Loop Closure	Forward Loop Closures	Backward Loop Closures
KITTI00	4541	Y	5	0
KITTI02	4661	Y	2	1
KITTI05	2761	Y	3	0

**Table 3:** Overview of computer configuration parameters.

Parameter Item	Specific Configuration
System version	Windows11
Processor	Inter CORE i7-12700H 4.7GHz
RAM	32.0 GB
Graphics card	NVIDIA 3060Ti
Simulation environment	Matlab R2021b

**Table 4:** $F_{1}$ scores of each algorithm on the KITTI dataset.

Sequence	ISC	M2DP	ESF	Ours
KITTI00	0.8676	0.9258	0.4613	0.9854
KITTI02	0.8257	0.7842	0.5913	0.8961
KITTI05	0.8526	0.8197	0.4997	0.9215

**Table 5:** Comparison of runtime on sequences 00 and 02 under different numbers of robots (unit: s).

Sequence	Number of Robots	RBCD	Ours
KITTI00	3	274.51	221.78
	5	183.93	133.06
	9	104.76	73.93
	20	53.22	33.26
	40	34.77	16.63
KITTI02	3	391.23	301.96
	5	211.38	181.17
	9	124.51	100.65
	20	61.67	45.29
	40	27.34	22.64

**Table 6:** Detailed information of the KITTI dataset.

Sequence	Environment	Trajectory Length (m)		
		Robot1	Robot2	Robot3
05	City	654	750	809
08	City	932	1398	1741

**Table 7:** Comparison of the trajectory error data for different algorithms based on the KITTI dataset (unit: m).

Sequence	Algorithms	ATE		
		Robot1	Robot2	Robot3
05	DiSCo-SLAM	Failed		
	DCL-SLAM	0.65	1.15	1.43
	Ours	0.24	0.36	1.05
08	DiSCo-SLAM	Failed		
	DCL-SLAM	3.07	4.35	4.56
	Ours	3.01	4.17	3.94

**Table 8:** Trajectory length information of each robot in the custom test dataset.

Sequence	Trajectory Length (m)	
	Robot1	Robot2
Custom test dataset	417	313

**Table 9:** Comparison of trajectory error data for different algorithms based on the custom test dataset (unit: m).

Sequence	Algorithms	ATE	
		Robot1	Robot2
Custom test dataset	DiSCo-SLAM	Failed	
	DCL-SLAM	1.35	1.16
	Ours	0.87	0.81

## Data Availability

The original contributions presented in the study are included in the article material, and further inquiries can be directed to the corresponding author.
